# A *Plasmodium* cysteine protease required for efficient transition from the liver infection stage

**DOI:** 10.1371/journal.ppat.1008891

**Published:** 2020-09-21

**Authors:** Elyzana Dewi Putrianti, Anja Schmidt-Christensen, Volker Heussler, Kai Matuschewski, Alyssa Ingmundson

**Affiliations:** 1 Parasitology Unit, Max Planck Institute for Infection Biology, Berlin, Germany; 2 Metabolism of Microbial Pathogens, Robert Koch Institute, Berlin, Germany; 3 Department of Experimental Medical Science, Lund University, Lund, Sweden; 4 Bernhard Nocht Institute for Tropical Medicine, Hamburg, Germany; 5 Institute of Cell Biology, University of Bern, Bern, Switzerland; 6 Department of Molecular Parasitology, Institute of Biology, Humboldt University Berlin, Berlin, Germany; Pennsylvania State University University Park: Penn State, UNITED STATES

## Abstract

The transitions between developmental stages are critical points in the *Plasmodium* life cycle. The development of *Plasmodium* in the livers of their mammalian hosts bridges malaria transmission and the onset of clinical symptoms elicited by red blood cell infection. The egress of *Plasmodium* parasites from the liver must be a carefully orchestrated process to ensure a successful switch to the blood stage of infection. Cysteine protease activity is known to be required for liver-stage *Plasmodium* egress, but the crucial cysteine protease(s) remained unidentified. Here, we characterize a member of the papain-like cysteine protease family, *Plasmodium berghei* serine repeat antigen 4 (*Pb*SERA4), that is required for efficient initiation of blood-stage infection. Through the generation *Pb*SERA4-specific antisera and the creation of transgenic parasites expressing fluorescently tagged protein, we show that *Pb*SERA4 is expressed and proteolytically processed in the liver and blood stages of infection. Targeted disruption of *PbSERA4* results in viable and virulent blood-stage parasites. However, upon transmission from mosquitoes to mice, *Pbsera4(-)* parasites displayed a reduced capacity to initiate a new round of asexual blood-stage replication. Our results from cultured cells indicate that this defect results from an inability of the *Pb*SERA4-deficient parasites to egress efficiently from infected cells at the culmination of liver-stage development. Protection against infection with wildtype *P*. *berghei* could be generated in animals in which *Pbsera4(-)* parasites failed to establish infection. Our findings confirm that liver-stage merozoite release is an active process and demonstrate that this parasite-encoded cysteine protease contributes to parasite escape from the liver.

## Author summary

*Plasmodium* parasites cause over 200 million cases of malaria every year. When parasites are transmitted by mosquito bite, they initially colonize the liver before they move into the blood and cause disease. During successful transition from the liver into the blood, *Plasmodium* cloak themselves in host plasma membrane as they egress from the liver cells. Although some aspects of how *Plasmodium* exit their host hepatocytes appear unique, certain attributes are shared across diverse pathogens. For example, protease activity is required not only for multiple stages of *Plasmodium* exit, but is also involved in the egress of some bacteria and other protozoan. Here we characterize a protease in *Plasmodium berghei* that is expressed in the liver and conserved across *Plasmodium* species. Through gene targeting, we found *Pb*SERA4 is required for efficient egress of *Plasmodium* from the liver. In the absence of this protease the transition between the liver and blood stages of growth is prolonged due to inefficient parasite release from liver cells. These findings provide new insights into the function of a conserved *Plasmodium* protease and into the process of *Plasmodium* escape from the liver.

## Introduction

For a parasitic disease to be maintained in a population, each step of an often complex life cycle must be sustained, and successful transitions between these steps are essential. *Plasmodium* parasites, which are responsible for malaria, alternate between a vertebrate and an insect host. Inside the vertebrate hosts, *Plasmodium* parasites enter suitable target cells and proliferate within an intracellular compartment termed the parasitophorous vacuole [1, 2]. As a consequence, parasites have to actively enter and exit their host cell in order to progress in the life cycle. Pathogen exit from host cells is a complex series of events, typically involving both pathogen-encoded factors as well as host-derived proteins [3, 4].

A hallmark of the *Plasmodium* life cycle in the mammalian host is the initial obligatory parasite population expansion phase inside the mammalian liver. Prior to the pathogenic asexual parasite propagation in red blood cells, *Plasmodium* parasites infect hepatocytes, where they transform from the motile sporozoite transmission form into spherical trophozoites that initiate multiple rounds of closed mitosis and form thousands of first-generation liver-stage merozoites [5, 6]. In order to egress from the liver, these merozoites escape the parasitophorous vacuole to the host hepatocyte cytoplasm, which requires a *Plasmodium* phospholipase [7, 8]. Upon parasitophorous vacuole membrane breakdown, the host cell architecture is altered to allow the budding of host-cell-derived extracellular vesicles containing infectious merozoites [7, 9]. These so-called merosomes emerge directly into the liver sinusoids and arrest in the lung capillaries where merozoites are discharged and invade neighboring erythrocytes to commence blood infection [6], the exclusive cause of clinical malaria symptoms.

Inhibitor studies revealed a crucial role for cysteine protease activity in egress of either liver-stage or blood-stage merozoites [7, 10–13], but the identity of the essential liver-stage cysteine proteases remains undetermined. A family of papain-like proteases termed the serine-rich antigen (SERA) family is encoded by all *Plasmodium* species and includes members that are expressed in the liver stage of infection [10, 14]. Several of the SERA proteins are important for exit of *Plasmodium* at different stages of development. For example, the *Plasmodium berghei Pb*SERA5/ECP1 is required for egress of sporozoites from oocysts in the mosquito midgut [15], whereas the *Plasmodium falciparum Pf*SERA5 and *Pf*SERA6 are important for exit of merozoites from blood-stage schizonts [16, 17]

SERA family members have been shown to be processed by the *Plasmodium* subtilisin-like protease 1, SUB1, in the parasitophorous vacuole prior to parasite egress [18]. In infected erythrocytes, cleavage and activation of SERAs is triggered by secretion of SUB1 into the parasitophorous vacuole. This proteolytic cascade is followed by breakdown of the parasitophorous vacuole and eventual release of blood-stage merozoites [11, 12]. SUB1 is not only required for egress of blood-stage *Plasmodium*; *P*. *berghei* SUB1 was also shown to be essential for liver-stage parasite egress [19, 20]. One of the SERA genes that shows high transcript levels during late liver-stage development is PbSERA4 [10, 14]. *Pb*SERA4 is a cysteine-type SERA that is well conserved across the known rodent- and primate-infecting *Plasmodium* species [21]. Here, targeted *PbSERA4* gene disruption reveals a role for this putative protease in the process of *Plasmodium* escape from the liver.

## Results

### The *Plasmodium berghei* SERA4 cysteine protease is expressed at multiple stages in the parasite life cycle

**Fig 1:**
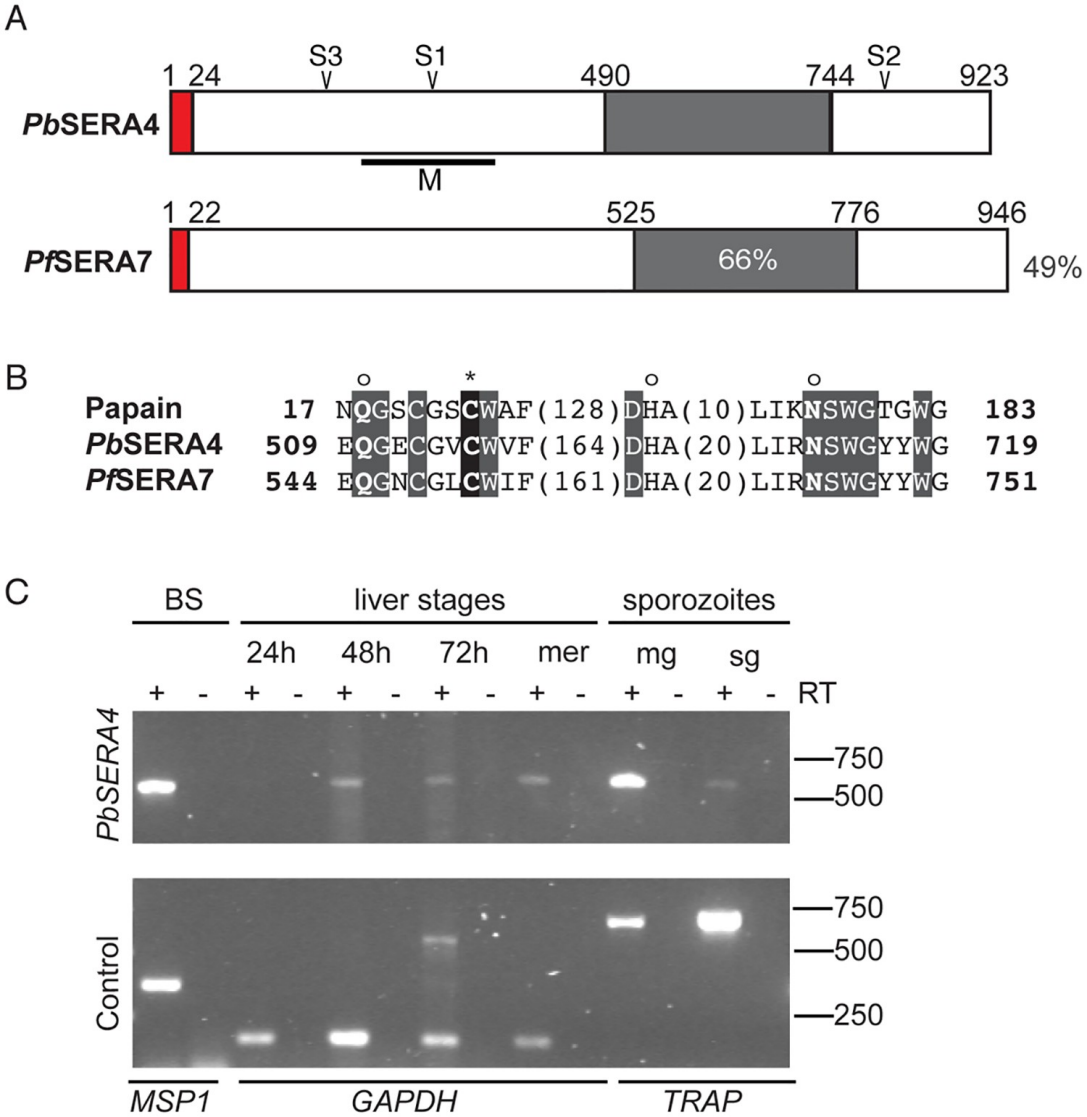
*Plasmodium berghei* SERA4 is a papain-like cysteine protease expressed across the *Plasmodium* life cycle. (A) Schematic structure of *P*. *berghei Pb*SERA4 (PBANKA_0304800) and its ortholog in *P*. *falciparum Pf*SERA7 (PF3D7_0207400). The putative signal sequences and the central papain-like cysteine protease domains are boxed in red and grey, respectively. Putative cleavage sites S1, S2 and S3 are as indicated at amino acids 295 (IKGEDDLD), 806 (ISGQTDNQ) and 173 (LTAKIEED), respectively. Antibodies were generated against the portions of *Pb*SERA4 indicated by the black line labeled M. Amino acid sequence identities between *Pb*SERA4 and *Pf*SERA7 are indicated as percent for both the overall sequence and the catalytic domain. (B) The catalytic residues of the papain family of cysteine proteases are conserved in *Pb*SERA4 and *Pf*SERA7. Strictly conserved amino acid residues are boxed in grey, and the putative active-site cysteine is boxed in black and marked with an asterisk. The catalytic histidine and flanking glutamine and asparagine residues, which form the oxyanion hole, are marked with an ‘o’. Papain is from *Carica papaya*. Bold numbers represent the amino acid positions within the protein before and after the catalytic domain; numbers in parenthesis represent the number of intervening amino acids at the indicated positions. (C) Expression profiling of *PbSERA4* in *P*. *berghei* ANKA. RT-PCR analysis from mixed blood stages (BS), liver stages (at 24, 48, and 72 hours after infection), liver stage merozoites (mer), and midgut (mg) and salivary gland (sg) sporozoites. Control transcripts were merozoite surface protein 1 (*MSP1*), glyceraldehyde dehydrogenase (*GAPDH*) or thrombospondin-related anonymous protein (*TRAP*) for blood, liver, and mosquito stages, respectively. Products run at the expected sizes in comparison to these products amplified from *P*. *berghei* genomic DNA (S1A Fig).

The *Plasmodium berghei PbSERA4* (PBANKA_0304800) gene encodes a cysteine-type SERA and is conserved and syntenic across the primate and rodent *Plasmodium* species [21]. The predicted orthologue of *PbSERA4* in *Plasmodium falciparum* is *PfSERA7* (PF3D7_0207400), and the proteins encoded by these genes are 44% identical and 57% similar (Fig 1A). Within the signature papain family cysteine protease domain (PFAM: PF00112), *Pb*SERA4 and *Pf*SERA7 share 66% amino acid identity (Fig 1A) and include the strictly conserved catalytic residues, *i*.*e*. an amino-terminal glutamine, the catalytic cysteine residue, a central histidine, and a carboxy-terminal asparagine (Fig 1B).

To profile *PbSERA4* expression throughout the *Plasmodium* life cycle, we employed standard RT-PCR analysis (Fig 1C and S1A Fig). *PbSERA4* transcripts are detectable in blood-stage *P*. *berghei* and can be detected in mid and late liver-stage parasites but are below the detection threshold in early liver stages. In the insect vector, *PbSERA4* transcripts can be detected in sporozoites isolated from the midguts or salivary glands of infected mosquitoes. Together, these data support our previous quantification of *PbSERA* transcripts in the mammalian host [10, 14] and confirm global transcript profiling data of both *P*. *berghei* [22] and *P*. *falciparum* [23–25]. Expression of *PbSERA4* in several developmental stages indicates functions during multiple points of the *Plasmodium* life cycle.

### Expression and localization of *Pb*SERA4 in late liver-stage *P*. *berghei*

**Fig 2:**
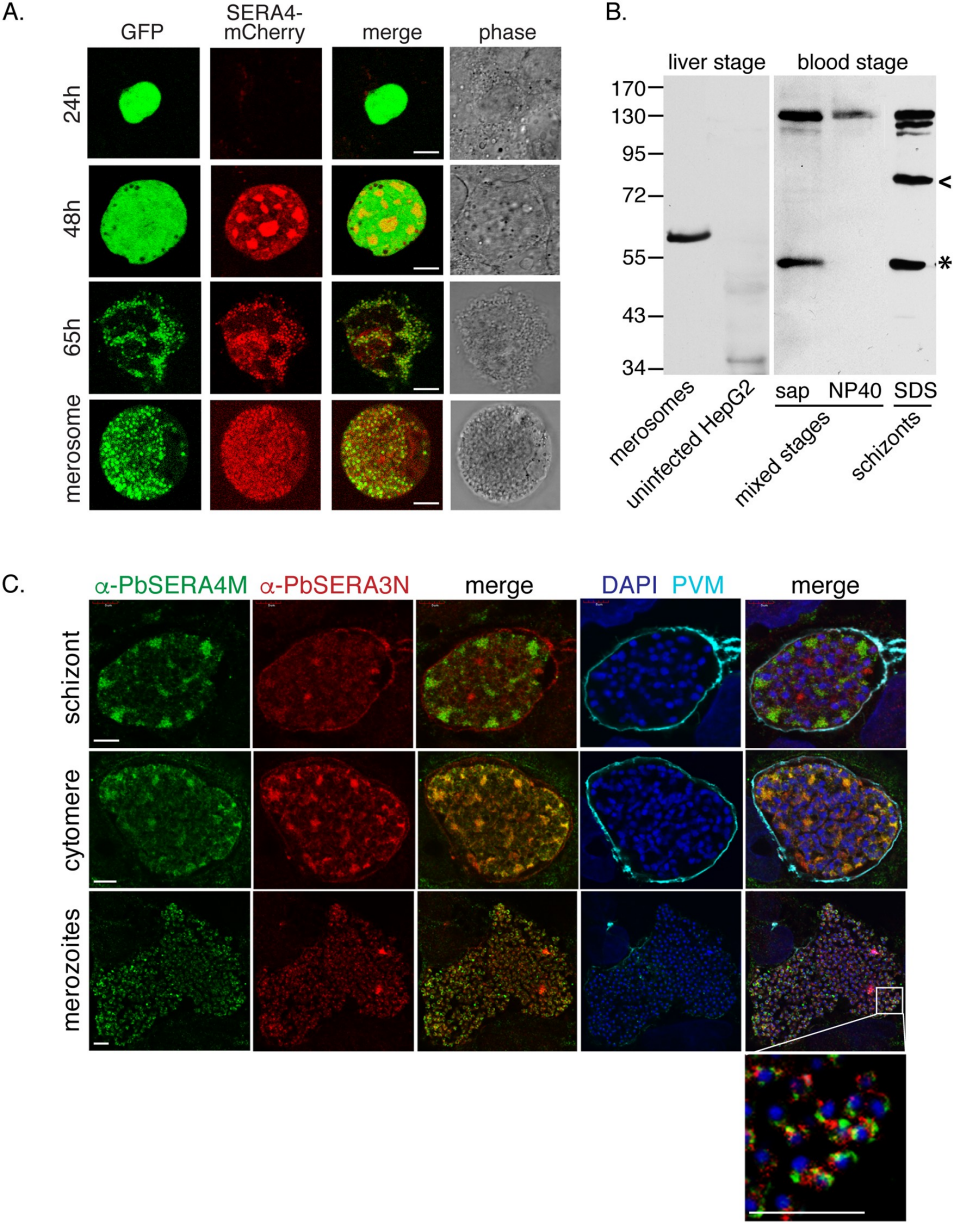
Proteolytic processing and localization of *Pb*SERA4 in late liver-stage *P*. *berghei*. (A) *Pb*SERA4-mCherry is detected in late liver-stage *P*. *berghei*. Huh7 hepatoma cells were infected with the *PbSERA4-mCherry P*. *berghei* ANKA line, fixed at the indicated time points and imaged by confocal microscopy. The parasites were detected with constitutive, cytoplasmic GFP expression (green). Scale bars, 8 μm. (B) The α-*Pb*SERA4M antibody detects full length *Pb*SERA4 and *Pb*SERA4 cleavage products. Liver-stage lysates were prepared from merosomes and detached cells (lane 1) collected from infected HepG2 cells and compared to uninfected HepG2 cells (lane 2). Proteins from mixed purified blood-stage parasites were initially solubilized with saponin and subsequently NP40 and compared to whole-cell SDS lysates of purified mature blood-stage schizonts. The predicted catalytic domain released by proteolytic processing is marked by the asterisk and appears to run slower in liver-stage compared to blood-stage samples. All displayed lanes are from one gel. (C) *P*. *berghei* ANKA-infected HepG2 cells were fixed at different time points (45-58h) post infection and stained with α-*Pb*SERA4M (green) in comparison to α-*Pb*SERA3N (red), α-*Pb*EXP1 (cyan), which labels the parasitophorous vacuole membrane (PVM), and the DNA dye DAPI (blue). For easier interpretation, images obtained with α-*Pb*SERA3N and α-*Pb*SERA4M were merged individually. Additional merges with images obtained with DAPI and α-*Pb*EXP1 are shown for orientation. Scale bar: 5μm.

To examine protein expression and assess the localization of *Pb*SERA4, we generated a transgenic *P*. *berghei* ANKA line that expresses fluorescently tagged *Pb*SERA4 from the native *PbSERA4* promoter (S1B Fig). Diagnostic PCR confirmed the integration of the targeting vector, which results in C-terminal tagging of *PbSERA4* with mCherry (S1C Fig). We then followed the life cycle progression of the resulting transgenic *PbSERA4-mCherry* parasite line, which expresses both constitutive cytoplasmic green fluorescence [26], and the red fluorescently tagged *Pb*SERA4 protein. *PbSERA4-mCherry* parasites progressed normally through the *Plasmodium* life cycle, including transmission to *Anopheles* mosquitoes and back to mice, indicating that expression of endogenously tagged *Pb*SERA4 is compatible with parasite viability in all stages. Consistent with reported transcriptome data [27, 28], we detected *Pb*SERA4-mCherry signal in late asexual blood stages and gametocytes (S1D Fig), and we could observe red fluorescence throughout exflagellation of male gametes. We failed to detect a red fluorescent signal in *PbSERA4-mCherry* sporozoites isolated from mosquito midguts or salivary glands despite the presence of *PbSERA4* transcripts (Fig 1C)[25]. We could confirm the absence of protein expression in sporozoites using a parasite line we generated in which the *PbSERA4* promoter drives GFP expression (S2 Fig). In these parasites, low levels of GFP are detected in oocysts but not in sporozoites isolated from salivary glands. In contrast, GFP expressed under control of the promoter of *PbSERA5*, which is known to function in the egress of sporozoites from oocysts, is clearly detectable in sporozoites. Neither GFP expressed from the *PbSERA4* promoter (S2 Fig), nor mCherry signal in *PbSERA4-mCherry* parasites (Fig 2A) could be detected in early liver-stage *P*. *berghei*. However fluorescent protein expression in both lines is evident later in the liver stage. *Pb*SERA4-mCherry could be detected 48 hours after infection of cultured cells with *PbSERA4-mCherry* sporozoites (Fig 2A). The *Pb*SERA4-mCherry signal progressively increased, and late liver schizonts and liver merozoites exhibited bright fluorescence.

In parallel to this tagging approach, we generated an antibody against the central domain of *Pb*SERA4 (α-*Pb*SERA4M) (Fig 1A). Because the central region of the SERA proteins is relatively conserved, we tested the specificity of the α-*Pb*SERA4M antiserum against recombinant purified central domains of the *P*. *berghei* proteins *Pb*SERA1-4 and found α-*Pb*SERA4M to exclusively recognize *Pb*SERA4 (S3 Fig). When lysates of liver-stage and blood-stage *P*. *berghei* were probed by western blot with α-*Pb*SERA4M, the detection of multiple fragments indicates that, like other SERA proteins, *Pb*SERA4 is proteolytically processed during infection. We predicted putative SUB1 cleavage sites within *Pb*SERA4 based on the cleavage pattern of other SERA proteins, and on the previously reported consensus recognition motif of SUB1 [18] (Fig 1A). In addition to the full-length protein, which is predicted to be 107kDa, the α-*Pb*SERA4M antibody recognizes a protein in the lysates of blood-stage schizonts that may represent the central catalytic domain, which is predicted to be 59kDa, as well as an intermediate cleavage product. If the order of *Pb*SERA4 processing occurs as described for *Pf*SERA5 [29, 30], we would expect this intermediate cleavage product to represent the product C-terminal to the predicted S1 cleavage site and have the predicted size of ~73 kDa. The intermediate cleavage product we detected corresponds to a slightly larger size (Fig 2B and S4C Fig, arrowhead) and could represent the N-terminal product that would be generated by cleavage only at the S2 cleavage site.

Proteolytic processing of other *Plasmodium* SERAs has been shown to occur in the parasitophorous vacuole prior to schizont rupture [18, 31]. To biochemically distinguish the parasitophorous vacuole from the parasite, purified mixed blood stages, which comprises mostly trophozoites, were treated with saponin to lyse the red blood cell and parasitophorous vacuole (Fig 2B). The putative catalytic domain-containing cleavage product appears in the saponin-soluble fraction, whereas the lysate of the remaining parasite-containing fraction contained only the full-length *Pb*SERA4 protein. This data suggests that in blood-stage *P*. *berghei*, the proteolytic processing of *Pb*SERA4 occurs in the parasitophorous vacuole. We also probed lysates from merosomes generated from sporozoite-infected hepatoma cells (Fig 2B). Here, only one band of approximately 60kDa is detected. In contrast to the sample containing blood-stage schizonts, we did not detect full length *Pb*SERA4 protein. This result may be due to greater enrichment of late-stage parasites in the merosome sample in comparison to the blood-stage schizont sample, which contains late trophozoites and early schizonts. The band detected in the merosomes appears to run higher on the gel than the putative *Pb*SERA4 proteolytic domain from the blood-stage parasites, which may be due to different properties of the samples or may indicate modifications or alternate processing. We were unable to detect protein with the α-*Pb*SERA4M antibody in late liver stage-infected cells, likely due to the low infection rate relative to the sensitivity of the antibody. Still, these results suggest that *Pb*SERA4 likely undergoes proteolytic processing by the end of the liver infection stage.

Using this antibody for immunofluorescence allowed detection of PbSERA4 in late liver-stage *P*. *berghei* and direct comparison to the localization of *Pb*SERA3, which is also abundantly expressed during late liver infection [10] (Fig 2C). In *P*. *berghei*-infected hepatoma cells the α-*Pb*SERA4M antibody recognized distinct regions in liver-stage schizonts, and in cytomeres the two signals overlapped in the parasite. Upon release of parasites from the parasitophorous vacuole, both *Pb*SERA4 and *Pb*SERA3 are found inside the first-generation merozoites with distinct localizations (Fig 2C). While *Pb*SERA3 is also detected surrounding the parasite in the parasitophorous vacuole, the α-*Pb*SERA4M signal appears restricted to the parasite. Together our findings show that *Pb*SERA4 is expressed and proteolytically processed in the liver and blood stages of infection and that protein expression is limited to the intermediate vertebrate host.

### Absence of *Pb*SERA4 delays the onset of blood-stage infection

**Fig 3:**
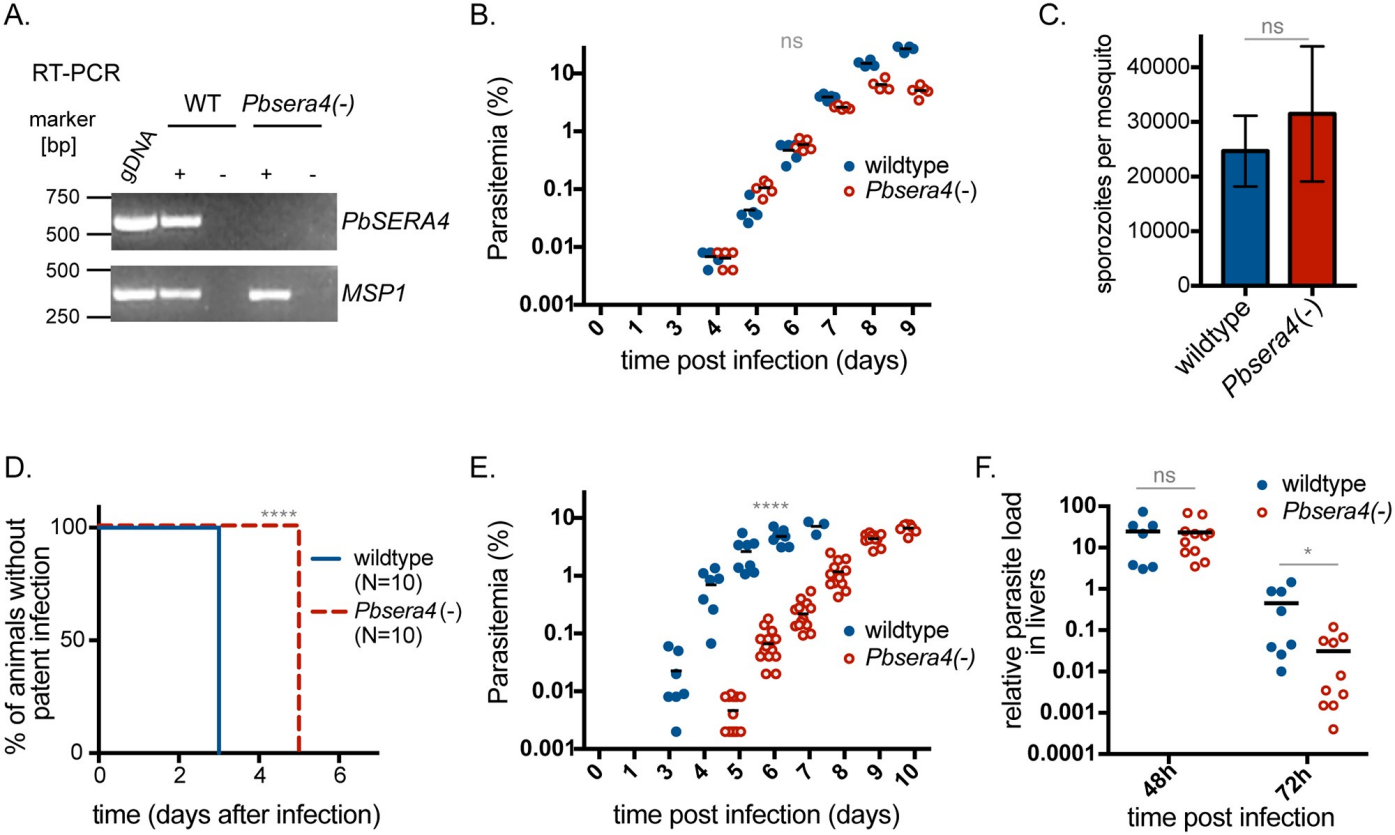
Absence of *Pb*SERA4 delays the time to appearance of *Plasmodium*-infected red blood cells. (A) Depletion of *PbSERA4* transcripts in *Pbsera4(-)-*ANKA parasites. cDNA from WT and *Pbsera4(-)* late-stage schizonts were used to verify the *SERA4* specific PCR reaction. *MSP1* transcript was used as a positive control. RT-PCR was performed in the presence (+) or absence (-) of reverse transcriptase (RT). Parasite genomic DNA (gDNA) was included as an amplification control. (B) Blood stage growth of *Pbsera4(-)-*ANKA *P*. *berghei* parasites. Naïve NMRI mice (n = 5) were injected intravenously with 1,000 wildtype or 1,000 *Pbsera4(-) P*. *berghei*-infected erythrocytes. Infection was then monitored by daily examination of Giemsa-stained blood smears to determine the parasitemia. ns, not significant (unpaired t-test, day 6 values). (C) Development of *Pbsera4(-)*-ANKA *P*. *berghei* in mosquitoes. Sporozoites extracted from salivary glands of infected mosquitoes were quantified 17–25 days after infection. Data is presented as the mean +/- SD. ns, not significant (paired t-test, *n* = 4 independent experiments). (D and E) Delayed patency following *Pbsera4(-)*-ANKA sporozoite infection. C57BL/6 mice were infected intravenously with 10,000 sporozoites, and Giemsa-stained blood smears were monitored daily to determine presence of parasites (D) ****, p<0.0001, Log-rank (Mantel-Cox) test, n = 10) and parasitemia (E) ****, p <0.0001 (unpaired *t* test, day 6 values). (F) *Pbsera4(-)*-ANKA parasites exhibit no growth defects in infected livers. RNA was extracted from livers of C57BL/6 mice infected with 50,000 to 100,000 sporozoites at the indicated time points. Levels of *Pb18s* rRNA were normalized to levels of mouse *HPRT* RNA by qPCR. ns, not significant; *, p < 0.05 (unpaired *t*-test).

To analyze the contribution of *Pb*SERA4 to parasite development and life cycle progression, we generated a *Pbsera4* knockout line. In a *P*. *berghei* ANKA line constitutively expressing GFP [26] the *PbSERA4* gene was replaced by the *Tgdhfr/ts* positive selection cassette through double homologous recombination (S4A Fig). After pyrimethamine-driven selection and cloning by limited dilution, the gene deletion in the *Pbsera4(*-*)* line was confirmed by diagnostic PCR, which showed the expected alteration of the genomic DNA (S4B Fig), RT-PCR, which confirmed absence of *PbSERA4* transcripts (Fig 3A), and western blot, which verified absence of the *Pb*SERA4 protein (S4C Fig).

Successful gene deletion indicated that although *PbSERA4* transcripts were detected at high levels during late blood stage development, *Pb*SERA4 is not essential for asexual blood-stage development. The effect of *Pb*SERA4 deletion on blood-stage growth was specifically tested by initiating infection of NMRI mice with intravenous delivery of infected red blood cells (Fig 3B). The time until appearance of infected cells in the blood and the growth rate of the *Pbsera4(-)* line were indistinguishable from wildtype *P*. *berghei*. Gametocytes were readily detected in the blood from mice infected with the *Pbsera4(-)* line, and we observed no defect in transmission of this line to mosquitoes, as evidenced by high numbers of sporozoites in the salivary glands of *Pbsera4(-)*-infected mosquitoes (Fig 3C). These findings indicate that *Pb*SERA4 is dispensable for *Plasmodium* blood-stage development as well as transmission to and sporogony in the mosquito vector.

To assess the impact that absence of PbSERA4 has on early stages of *P*. *berghei* development in mammalian hosts, C57BL/6 mice were injected intravenously with 10,000 *Pbsera4(-)* sporozoites. While wildtype-infected red blood cells appear in the blood three days after infection with sporozoites, *Pbsera4(-)* parasites did not appear in blood smears until five days after infection (Fig 3D; S2 Table). After appearance in the blood, *Pbsera4(-)* parasites grew at rates comparable to wildtype *P*. *berghei* (Fig 3E). Infection using 10x more sporozoites or infection of SD rats with *Pbsera4(-)* sporozoites resulted in similar, approximately two-day, delays in appearance of blood-stage infection in comparison to infections with wildtype sporozoites (S2 Table).

To determine whether this delay in patency observed upon infection with *Pbsera4(-)* sporozoites is due to inefficient infection of hepatocytes or reduced development of parasites in the liver, we assessed the parasite burden in the livers of sporozoite-infected C57BL/6 mice using quantitative PCR (Fig 3F). *Pbsera4(-)* parasites reached levels equivalent to wildtype parasites in livers of infected animals 48 hours after infection. The parasite burden in livers infected by wildtype parasites drops by 72 hours after infection due to egress and merozoite release. Although the appearance of *Pbsera4(-)* parasites in the blood of sporozoite-infected animals is delayed, the *Pbsera4(*-*)* parasites do not appear to be retained in the liver at 72 hours after infection. The higher levels of detectable parasite RNA in livers of wildtype-infected animals at 72 hours post-infection in comparison to *Pbsera4(-)*-infected animals is likely due to contamination with blood-stage parasites that have grown in the blood of wildtype-infected individuals by 72 hours post-infection. The comparable parasite burden near the onset of egress, however, indicates that the delay in time to patency occurring during *Pbsera4(-)* infection is not due to reduced growth of the parasites in the liver.

### *PbSERA4* is required for efficient egress of liver-stage *P*. *berghei*

**Fig 4:**
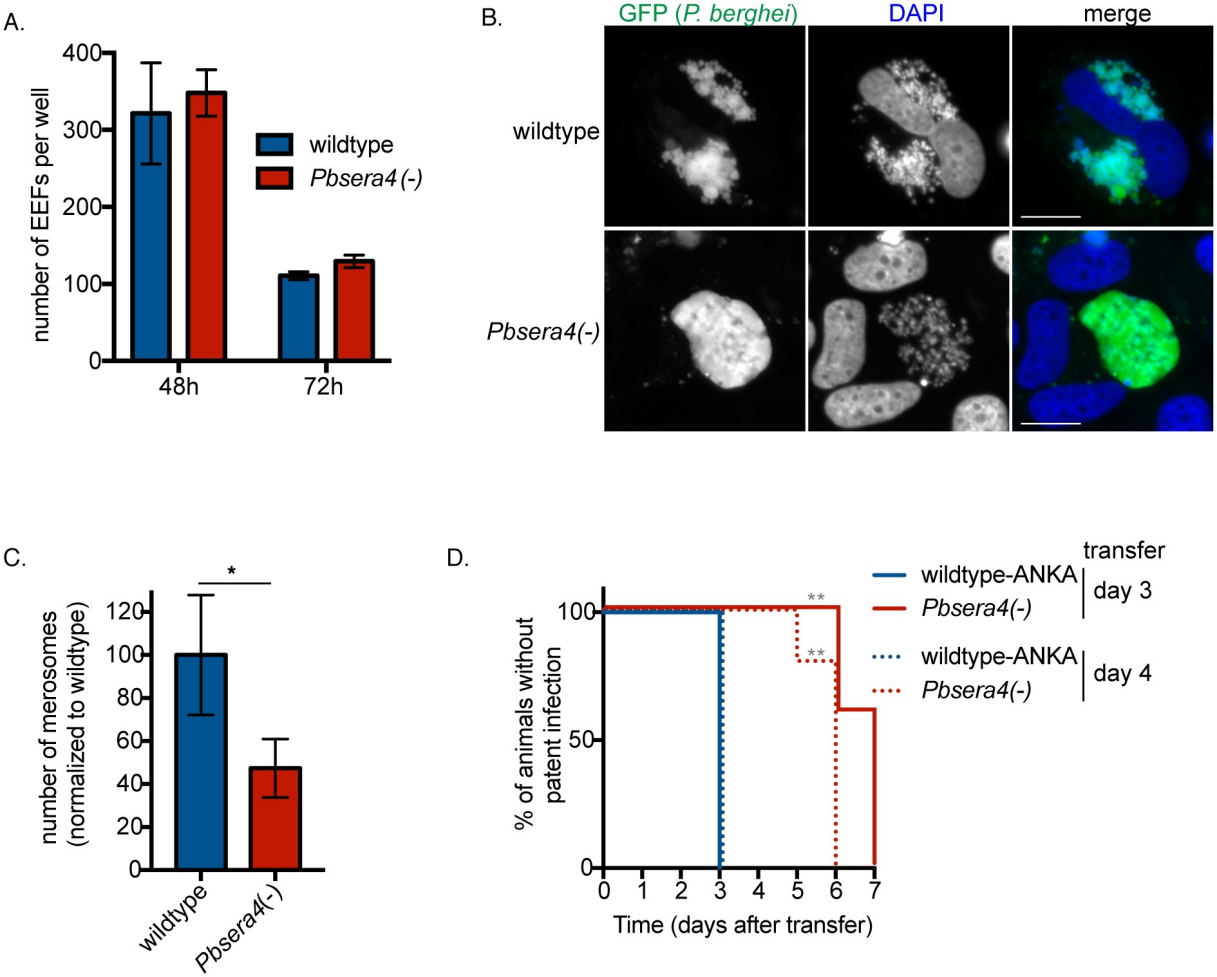
*Pb*SERA4 is not required for liver-stage growth, but is needed for efficient egress from cultured cells. (A) Huh7 cells were infected with sporozoites of the indicated *P*. *berghei* ANKA lines and fixed at the indicated time points. *P*. *berghei* exoerythrocytic forms (EEF) were visualized and quantified by fluorescence microscopy. Data is presented as the mean +/- SD. (B) Infected Huh7 cells were fixed and imaged at 65 hours post-infection. At least 50 cells infected with each strain were visualized. More than a quarter of wildtype EEFs were at the advanced stage shown in the upper panel, whereas in the *Pbsera4(-)*-infected cells, no individual merozoites could be detected in any cell, and none of the *Pbsera4(-)* parasites had progressed further than the cytomere stage. (C) Merosomes and parasite-containing detached cells were collected from the supernatants of infected Huh7 cells between 65 and 72 hours of liver-stage infection and quantified. Data compiled from five experiments are shown and presented as means +/- SEM. * P<0.05 (ratio paired two-tailed *t*-test, n = 5 independent experiments). (D) Sub-inoculation from pre-patent *Pbsera4(-)*-ANKA infected animals. C57BL/6 mice were infected intravenously with 10,000 wildtype-ANKA or *Pbsera4(-)-*ANKA sporozoites. Blood was transferred from these infected mice to naïve NMRI mice at either 3 or 4 days after infection, and Giemsa-stained blood smears were monitored daily to determine presence of parasites. **, p<0.01, (Log-rank (Mantel-Cox) test, n = 5).

To further investigate the stage-conversion phenotype we see in *Pbsera4(-)*-infected mice, we confirmed the ability of *Pbsera4(-)-*ANKA sporozoites to efficiently infect and grow in cells in *in vitro* infection experiments (Fig 4). When Huh7 cells were infected, *Pbsera4(-)* sporozoites developed into exo-erythrocytic forms (EEF) in numbers similar to those formed by wildtype sporozoites (Fig 4A). Imaging of infected cells late in liver-stage development revealed that while several wildtype parasites existed as merozoites that were detaching from one another and were within the host cell cytoplasm, the *Pbsera4(-)* parasites that were imaged appeared to remain as cytomeres (Fig 4B). When merosomes and parasite-containing detached cells were collected from the supernatants of infected cultured cells following liver-stage infection, notably fewer *Pbsera4(-)* merosomes were detected (Fig 4C, S3 Table). *Pbsera4(-)*-infected cultures generated approximately half the numbers of merosomes produced by wildtype-infected cultures. When equal numbers of wildtype or *Pbsera4(-)* merosomes produced by cultured cells were injected into mice, all mice developed a blood-stage infection (S3 Table), indicating that the *Pbsera4(-)* liver-stage merozoites that are generated are as capable of infecting red blood cells and establishing a blood-stage infection as wildtype merozoites. Together, these data indicate that liver-stage *Pbsera4(-)* parasites are released inefficiently from infected cells, but that they are infectious to red blood cells.

If liver-stage merozoite egress is impeded *in vivo*, this could manifest either as a reduction in the numbers of merozoites released from the liver or a delay in their release, and either could cause the delay in the onset of blood-stage infection we observed in infected mice. To distinguish between these two possibilities, we transferred blood from sporozoite-infected animals into naïve animals three days or four days after infection. At these time points, although patent infection is not yet visible in blood smears of *Pbsera4(-)*-infected animals, all animals that received the blood sub-inoculation developed a blood-stage infection (Fig 4D). This indicates that merozoites have been released from the livers by this time, but that blood-stage infection is below the level of detection by Giemsa-stained blood smears. Blood-stage parasites did not appear in the *Pbsera4(-)-*sub-inoculated animals until three days after the appearance of blood-stage parasites in animals receiving the wildtype sub-inoculation. These results illustrate that the numbers of blood-stage parasites present at this time in the *Pbsera4(-)*-sporozoite infected animals is remarkably fewer in comparison to wildtype-sporozoite-infected animals. These data together with the observed decrease in detectable parasite RNA in the livers of *Pbsera4(-)*-infected animals at three days post-infection (Fig 3F) suggest that the timing of liver-stage egress is not markedly delayed in a *Pbsera4(-)* infection, but rather that only few liver-stage merozoites reach the blood.

### Absence of *PbSERA4* prolongs the prepatent period of the NK65 *P*. *berghei* strain and leads to the upregulation of *PbSERA3* during liver stage development

**Fig 5:**
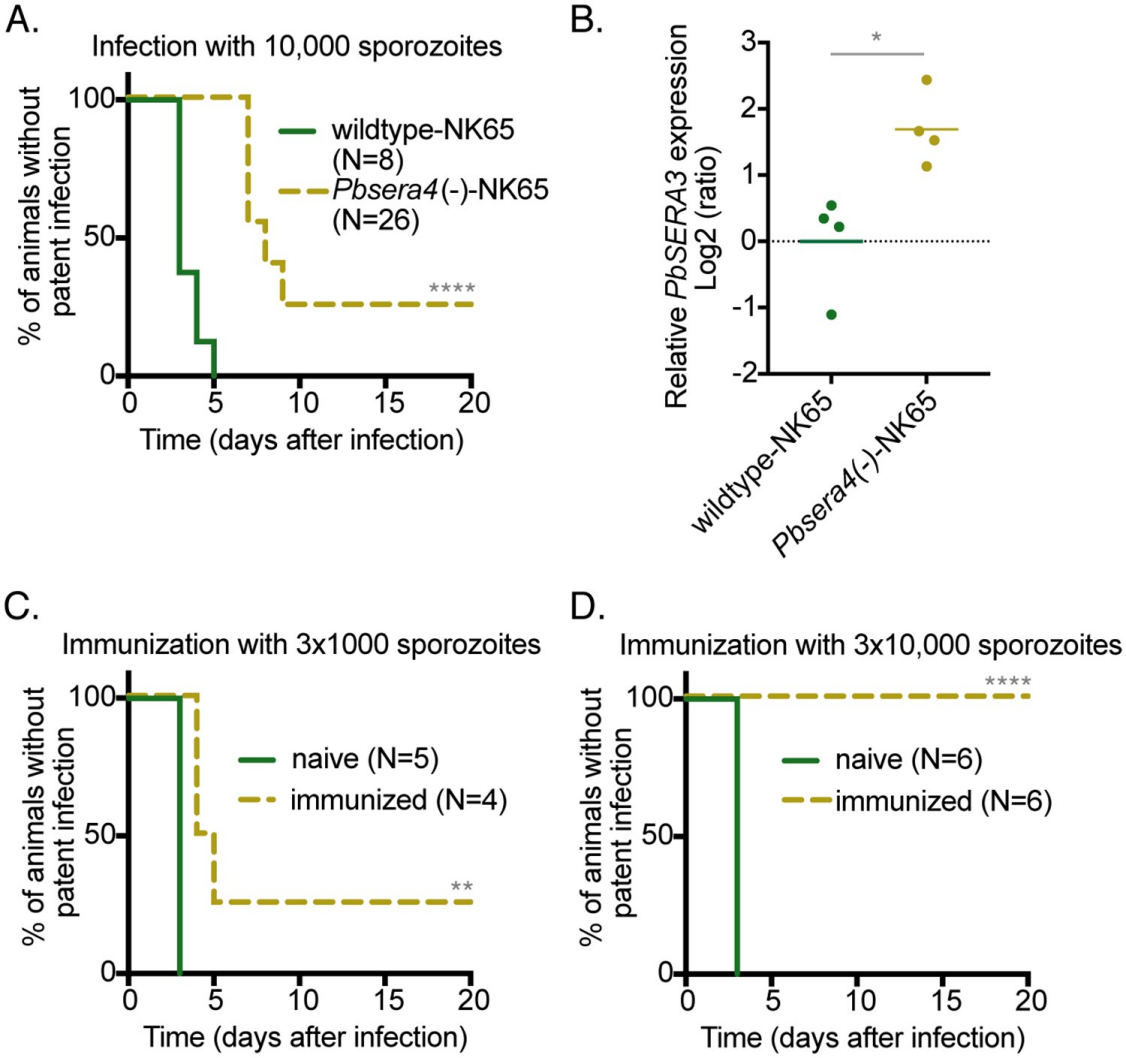
*Pbsera4(-)*-NK65 parasites inefficiently initiate blood-stage infection and can confer protection against sporozoite challenge infection. (A) Delayed patency following *Pbsera4*(-)-NK65 sporozoite infection. C57BL/6 mice were infected intravenously with 10,000 sporozoites, and Giemsa-stained blood smears were monitored daily to determine the presence of parasites. ****, p<0.0001, Log-rank (Mantel-Cox) test. (B) *PbSERA3* is upregulated in the liver in the absence of *PbSERA4*. PbSERA3 gene expression was analyzed in livers of C57BL/6 mice two days after infection with wildtype and *Pbsera4*(-)-NK65 sporozoites. Relative gene expression was normalized to *Pb18s* and the average of the wildtype-NK65 samples using the Pfaffl method. *, p<0.05 (unpaired t test, n = 4). (C and D) C57BL/6 mice were immunized three times with 1,000 or 10,000 *Pbsera4(-)*-NK65 sporozoites and were challenged two months after the last immunization with wildtype NK65 sporozoites. Giemsa-stained blood smears were monitored daily to determine the presence of parasites. **, p<0.01; ****, p<0.0001, Log-rank (Mantel-Cox) test.

To confirm that *Pb*SERA4 is required for efficient *P*. *berghei* stage conversion from liver- to blood-stage growth, we generated a second knockout line in the NK65 strain of *P*. *berghei* using the same approach we used for the knockout line generated in the ANKA strain (S4D Fig). When sporozoites of the resulting *Pbsera4(-)*-NK65 line were used to infect mice, the *Pbsera4(-)*-NK65 parasites not only took longer to appear in the blood of infected animals in comparison to wildtype NK65 parasites, but some animals infected with the knockout line never developed a blood-stage infection (Fig 5A). The four-day delay in time to patent blood-stage infection in the absence of *PbSERA4* was likewise seen in *P*. *berghei* NK65-infected SD rats and also following infection by mosquito bite (S2 Table).

Targeted deletion of *SERA* genes has been shown to influence the expression of other *SERA* genes [14, 32]. Absence of the serine-type *PfSERA4* leads to upregulation of the essential serine-type *PfSERA5* [32], and deletion of the serine-type *PbSERA1* and *PbSERA2* results in upregulation of cysteine-type *PbSERA3* [14]. We, therefore, examined the expression of the other liver stage-expressed cysteine-type SERA, *PbSERA3*, in the absence of *Pb*SERA4 at the end of liver-stage development (Fig 5B). *PbSERA3* is upregulated in *Pbsera4(-)*-NK65 in comparison to the wildtype line in the livers of sporozoite-infected animals. Altering the expression of *PbSERA3* may represent a compensatory mechanism by the parasite and may contribute to the emergence of parasites in the blood in the absence of *PbSERA4*.

### Infection with *Pb*SERA4-deficient NK65 *P*. *berghei* sporozoites can confer protection against reinfection

Following our observation that some mice stayed malaria-free once infected with either 1,000 or 10,000 *Pbsera4(-)*-NK65 sporozoites, we used these mice to test whether immunization with *Pbsera4(-)-*NK65 sporozoites can confer sterile protection against challenge with wildtype NK65 sporozoites. These protection assays could not be performed with the *Pbsera4(-)-*ANKA line because all animals infected with this line developed a blood-stage infection, albeit delayed. The two groups of mice (N = 10) were immunized with another two doses of either 1,000 or 10,000 *Pbsera4(-)*-NK65 sporozoites, respectively (S4 Table, Fig 5C and 5D). Half of the immunized mice were challenged by infection with wildtype sporozoites two months after the last immunization. All mice immunized with 10,000 *Pbsera4(-)*-NK65 sporozoites were protected and did not develop detectable blood-stage infections (Fig 5D). The mice immunized with low doses of 1,000 *Pbsera4(-)*-NK65 sporozoites were modestly protected; three mice of four developed a delayed blood-stage infection when challenged two months after the last immunization (Fig 5C).

To assess long-term protection, the remaining immunized mice were challenged by injection of wildtype sporozoites 20 months after the last immunization (S4 Table). The six mice that had shown protection after challenge at 2 months were also re-challenged at this time. None of the mice that had received only three immunizations were protected at 20 months; blood-stage parasites were detected in all of these animals after challenge. In contrast, all five mice that received immunization doses of 10,000 *Pbsera4(-)*-NK65 sporozoites and were protected from the challenge at two months, were also protected against the re-challenge infection at 20 months. This result indicates that the fourth exposure to pre-erythrocytic *P*. *berghei* conferred long-lasting protection in these mice.

Our results with the NK65 knockout line confirm that the absence of *Pb*SERA4 interferes with the parasites’ ability to establish blood-stage infection. We further demonstrate that although protection wanes over time, immunization with *Pbsera4(-)*-NK65 sporozoites can confer sterile protection.

## Discussion

Our studies have identified a role for *Pb*SERA4 in the escape of *Plasmodium* parasites from the liver stage of infection. All functional data on the *Plasmodium* SERA family thus far indicate roles for these proteases in parasite egress [15, 17, 33]. Until now, of the *Plasmodium SERA* family, only the contributions of *PbSERA1* and *PbSERA2* to liver-stage egress have been tested [14]. Neither of these serine-type SERAs are necessary for *Plasmodium* exit from the liver [14], and the specific cysteine proteases known to be needed for liver-stage egress [7] have remained unidentified. The central finding of this study is that loss of *Pb*SERA4 function reduces the efficiency of liver-stage egress and weakens the transition from liver-stage growth to blood-stage infection.

*Pb*SERA4 is one of the cysteine-type SERAs, meaning that unlike the serine-type SERAs, the papain-like catalytic site is intact and includes all key catalytic amino acid residues [21, 34]. The cysteine-type *SERA* genes have clear orthologs across *Plasmodium* species and the cysteine-type SERA we describe here appears to have emerged in a common ancestor to all primate- and rodent-infecting *Plasmodium* [21]. The predicted ortholog of *PbSERA4* in *P*. *falciparum* is *PfSERA7*, which is syntenic with *PbSERA4* and shares the highest sequence identity with *PbSERA4* of the *P*. *falciparum SERA* genes. Furthermore, *PfSERA7* has been shown to be dispensable for blood-stage *P*. *falciparum* growth [16], which is consistent with our data showing the *PbSERA4(-) P*. *berghei* lines grow proficiently in the blood.

Our expression and localization studies of *Pb*SERA4 using a specific antibody and a parasite line in which the endogenously encoded protein is fluorescently tagged show that *Pb*SERA4 is expressed in blood-stage and late-liver-stage *Plasmodium*. Previous studies demonstrated that SERA family members in *P*. *falciparum* and *P*. *berghei* are proteolytically processed to their active form [10, 18, 31, 33], and our data demonstrate that *Pb*SERA4 appears also to be proteolytically processed during infection. Processing of at least some SERA family members occurs in the parasitophorous vacuole upon secretion of SUB1 [18]. In contrast to other liver-stage-expressed SERAs [10, 14], *Pb*SERA4 was only detected inside the parasite, but the localization in liver-stage schizonts detected by both antibody staining and fluorescent tagging highly resembles parasite endoplasmic reticulum [35]. This localization and the predicted N-terminal signal peptide suggest that *Pb*SERA4 is engaged by the parasites’ secretory pathway. The region of *Pb*SERA4 used to generate the α-*Pb*SERA4M antibody contains a putative SUB1 cleavage site; therefore, there is a possibility that this antibody may only recognize uncleaved protein when the protein is in its native state after PFA fixation. Thus, if PbSERA4 cleavage products are in the parasitophorous vacuole during liver infection, the antibody may have failed to detect them.

Targeted deletion of *PbSERA4* hindered the ability of *P*. *berghei* to transition from the liver into the blood of infected animals. We observed that time until the appearance of blood-stage parasites in animals following liver-stage infection increased in the absence of *Pb*SERA4. The length of this time delay depended on the *P*. *berghei* strain. The *Pbsera4(-)*-NK65 line was more severely impaired than the *Pbsera4(-)*-ANKA line; patent infection was delayed by approximately four days with the NK65 knockout line and two days with the ANKA knockout line. In some animals, the *Pbsera4(-)*-NK65 line even failed to establish blood-stage infection. *Pbsera4(-)* liver-stage growth was unobstructed either *in vivo* or in cultured cells. Furthermore, blood-stage growth of *Pbsera4(-)* parasites proceeded with normal growth rates regardless of the route of infection. These results indicate that the restriction of *Pbsera4(-)* parasites occurs between these two growth stages. *Pbsera4(-)*-infected cultured cells produce fewer merosomes, which suggests a defect in egress of liver-stage merozoites from infected cells. This conclusion would be consistent with the known egress functions of other SERA family members. The levels of *Pbsera4(-)* parasite RNA in infected livers drop by 72 hours post-infection, indicating that parasite-containing cells do not remain detectable in the liver in spite of few merozoites reaching the bloodstream. An alternative possibility is that *Pb*SERA4 is required for egress of liver-stage merozoites from merosomes after they have entered the blood stream. *Pb*SERA4 is also expressed in blood-stage parasites, which is reported as well for *Pf*SERA7. Our data confirm that *Pb*SERA4 is not essential for asexual blood-stage development, as demonstrated for *Pf*SERA7 [16], and reveal that *Pb*SERA4 is also expendable for gametocyte egress, which was demonstrated by efficient infection of mosquitoes by the *Pbsera4(-)* lines. Whether *Pb*SERA4 contributes to egress with functions for which other factors can compensate in its absence remains to be determined.

The cysteine-type *SERA* genes form three phylogenetically distinct groups [36]. Group one, represented by *Pb*SERA5 and *Pf*SERA8, in *P*. *berghei* and *P*. *falciparum*, respectively, is required for egress of sporozoites from oocysts [15]. Group three, represented by *Pb*SERA3 and *Pf*SERA6, is essential for egress of merozoites from red blood cells [17]. *Pb*SERA4 is within group two, and our data suggests that this group contributes to egress of liver-stage merozoites from the liver. In contrast to the studied representatives of the other two groups, *Pb*SERA4 is not vital to the process of parasite egress. In the absence of *PbSERA4*, a few parasites escape the liver and successfully reach the blood. Another enzymatic protein that contributes to liver-stage *Plasmodium* egress, *P*. *berghei* phospholipase, is also not strictly required for parasite transition into the blood [8]. Because of the complexity and importance of this process for establishing an infection in the mammalian host, *Plasmodium* may have multiple mechanisms by which to promote its transition from the liver into the blood. The upregulation of *Pb*SERA3 in liver-stage *Pbsera4(-)-*NK65 parasites suggests that this other cysteine-type SERA may be able to, in part, compensate for the loss of *PbSERA4* in these parasites. While the localizations of *Pb*SERA3 and *Pb*SERA4 do not always overlap, they do colocalize in the parasite in the cytomere stage. While the function of *Pb*SERA3 in the liver remains undefined, together these data indicate that *Pb*SERA3 could play a role in the ability of *PbSERA4*-deficient parasites to transition to the blood and may therefore contribute to liver-stage egress in wildtype parasites as well.

Because multiple SERA proteins are expressed at the same time in both the blood and liver, it has been proposed that they may influence the function of one another. For example, one SERA could alter the function of another by affecting the recognition of its substrate or by influencing its activation by proteolytic processing [34]. While this has been proposed in particular for the serine-type SERA proteins, we cannot formally exclude this as a possibility for the cysteine-type SERAs. However, because *Pb*SERA3 is essential for blood-stage development, and processing of the corresponding *P*. *falciparum* ortholog, *Pf*SERA6 is essential for its activity [17], we suspect that the processing and function of *Pb*SERA3 is unaltered in parasites lacking *Pb*SERA4, at least in the blood stage of infection.

The failure of the *Pbsera4(-)*-NK65 line to establish blood-stage infection in some animals provided an opportunity to assess the ability of this parasite line to elicit an immune response that can protect animals against reinfection. Late-liver-stage-arresting *P*. *berghei* often confer strong protection against re-infection [37], but often present the risk of breakthrough infections. The ability of these parasites to develop into late liver stages and elicit protective responses indicates that perhaps this SERA gene could be deleted in combination with other genes to achieve iterative attenuation at multiple checkpoints in the formation of first-generation merozoites, and this approach could lead to a safe and efficacious whole sporozoite vaccine strategy.

Taken together, these studies have revealed a role for *Pb*SERA4 in exit of *Plasmodium* from the liver. While not strictly essential for stage-conversion, *Pb*SERA4 contributes to efficient transition between liver- and blood-stage growth. As in egress of blood-stage merozoites, a proteolytic cascade involving a distinct member, *Pb*SERA4/*Pf*SERA7, of the conserved family of papain-like cysteine proteases contributes to exit of merozoites from the liver.

## Materials and methods

### Ethics statement

All animal work was conducted in accordance with the German ‘Tierschutzgesetz in der Fassung vom 18. Mai 2006 (BGBl. I S. 1207)’, which implements directive 86/609/EEC from the European Union and the European Convention for the protection of vertebrate animals used for experimental and other scientific purposes. All appropriate measures were taken to reduce the pain or discomfort of the animals, and the protocol was approved by the ethics committee of the University of Heidelberg and Max Planck Institute for Infection Biology and by the state authorities (Regierungspräsidium Karlsruhe G154/05 and Landesamt für Gesundheit und Soziales G0469/09).

### Experimental animals, cells and parasite lines

Naval Medical Research Institute (NMRI) mice, C57BL/6 mice, and Sprague Dawley (SD) rats were purchased from Charles River Laboratories (Sulzfeld, Germany). *Anopheles stephensi* mosquitoes were raised under a 14 h light/ 10 h dark cycle, 75% humidity and at 28 °C (non-infected) or 20 °C (infected). The Huh7 hepatoma cell line was cultured in RPMI supplemented with 10% FCS, 100 U penicillin, 100 μg/ml streptomycin, 2 mM L-glutamine, 10 mM HEPES and 1× non-essential amino acids (NEAA) (Gibco). The HepG2 hepatoma cells were cultivated in EMEM (Gibco) containing 10% fetal calf serum, 1% L-Glutamine, 1% Penicillin/Streptomycin and 1% MEM Non-essential amino acids (PAA Laboratories GmbH, Pasching, Austria). Wild-type *P*. *berghei* were either the *P*. *berghei* ANKA strain, ANKA clone 507 [26], which expresses GFP, or *P*. *berghei* NK65 [38].

### Transcript detection and quantitation

Transcript detection in the various stages was performed as described [14]. RNA purification was followed by cDNA synthesis and products were amplified using specific primers described in S1 Table. Standard curves were generated for all primers using WT cDNA serial dilutions and gave amplification efficiencies of 90–100%. Relative *PbSERA3* gene expression was normalized to *Pb18s*, and calculated with the Pfaffl method to account for differing primer efficiencies [39]. The average Ct values from the wildtype-NK65-infected livers were used as calibrators in this analysis.

### Generation of *sera4(-)* and mCherry-tagged *SERA4* parasites

Transgenic *P*. *berghei* lines were generated as described previously [14, 26]. For mCherry tagging, the *PbSERA4* locus was amplified with primers mCherry-SERA4for and mCherry-SERA4rev and integrated into B3D+mCherry [40] in frame with the mCherry coding sequence. For targeted disruption of *PbSERA4*, a replacement vector was generated through amplification of genomic regions flanking *PbSERA4* using primers SERA4rep1for and SERA4rep2rev or SERA4rep3for and SERA4rep4rev and cloned into b3D.DT^H.^D (provided by Dr. Andrew Waters, Glasgow University) flanking the *Tgdhfr/TS* expression cassette. *P*. *berghei* parasites were transfected with linearized plasmids as described [14, 26], injected into naïve NMRI mice and selected by oral pyrimethamine. Integration of the constructs was confirmed by PCR with specific primer pairs (see supplementary information for details), and clonal lines were isolated via limiting dilution. All oligonucleotide sequences are listed in S1 Table.

### Generation of anti-*Pb*SERA4 antibodies

The central (M) (Phe^216^-His^365^) region of *Pb*SERA4 was amplified from *P*. *berghei* ANKA cDNA. The resulting PCR product was ligated into pGEX6P-1 (Amersham, Bucking-hampshire, England) and expressed in *Escherichia coli* BL21 cells (Stratagene) as glutathione *S*-transferase (GST) fusion proteins. Bacteria were lysed by sonication in PBS containing 10 mM EDTA and CompleteTM protease inhibitor (Roche Molecular Biochemicals). GST-fusion proteins were purified from the supernatant using glutathione-agarose as described by the manufacturer (Amersham Biosciences). 20μg of purified *Pb*SERA4-N-GST or *Pb*SERA4-M-GST fusion protein was used to immunize six week old female NMRI mice by intraperitoneal injection along with complete Freund adjuvant, followed by multiple boosting immunizations. For anti-PbSERA3N, blood samples were collected following immunization, to test serum reactivity against the SERA4-N protein. From a positive mouse spleen, cells were isolated and fused to the mouse myeloma cell line X63Ag8.653. Supernatants were screened by indirect ELISA and single-cell clones were isolated by limited dilution.

### Immunofluorescence analysis

For analysis of *Pb*SERA4 localization in late liver stages, infected HepG2 cells were fixed with 4% formaldehyde, permeabilized with ice-cold methanol and incubated with mouse anti-PbSERA4M serum, rat anti-PbSERA3N serum [10] (1:200 in 10% FCS diluted in PBS) or a chicken anti-Exp1 antibody. Bound antibodies were detected using fluorescently-conjugated secondary antibodies (Molecular Probes, Leiden, The Netherlands). Labeled cells were examined by confocal microscopy using the Olympus FV1000 (SIM- scanner and spectral detection).

### SDS-PAGE and western blot analysis

Blood-stage-schizont-infected red blood cells were enriched on a 55% Nycodenz gradient and lysed in 0.15% saponin in PBS. Pelleted parasites were subsequently lysed in a TRIS-buffered solution containing 150mM NaCl and 1.0% NP-40. For the merosome sample, loaded lysates contained merosomes and detached cells collected from at least two wells of a 24-well dish containing infected HepG2 cells. Merosomes and detached cells were lysed directly in Laemmli sample buffer. Lysates were separated on 10% SDS-PAGE reducing gels and transferred to nitrocellulose membranes. Membranes were probed with mouse α-*Pb*SERA4-M antisera (1: 4000). Horseradish peroxidase-conjugated goat anti-mouse (1: 20.000, Pierce) were used for detection, and bands were visualized by enhanced chemiluminescence Pico or Femto Detection Kit (Pierce).

### Parasite phenotyping during *Plasmodium* life cycle progression

To quantify patency or blood stage development, daily Giemsa-stained blood smears of infected NMRI and C57BL/6 mice were counted. Exflagellation of microgametes were examined prior to mosquito feeding. For analysis of liver stage development, sporozoites were added to cultured hepatoma cells, either Huh7 or HepG2 as indicated, incubated for two hours at 37°C, and washed. After fixation, exoerythrocytic forms (EEFs) were visualized by their GFP expression or by using an anti-*Pb*HSP70 antibody [41]. Merosomes and parasite-containing detached cells were collected 65–70 hours post infection when merosomes were visible in the culture by light microscopy. To collect and quantify merosomes, the culture was gently agitated by gently tapping the plate, and the culture medium was collected and centrifuged for 3 minutes at 2000 rpm. The pellet was resuspended in a low volume and merosomes were quantified using a Neubauer chamber and fluorescence microscopy, which allowed identification based on parasite GFP fluorescence. The parasite burden of *Pbsera4(-)* and WT infected livers was determined as described [42, 43]. Briefly, C57Bl/6 mice were injected with 10^4^ sporozoites intravenously, and livers of infected animals were harvested at the indicated time points. Total RNA was extracted and relative parasite levels were determined with quantitative–PCR by comparing the mean C_*t*_ value of the *P*. *berghei 18s ribosomal subunit* to the mean C_*t*_ value of the *Mus musculus* hypoxanthine phosphoribosyl transferase (*HPRT*) in the generated cDNA. Subinoculation was performed through transfer of blood taken from mice at day three or four after sporozoite inoculation. Blood from infected mice was transferred into naïve mice by intravenous injection. About 100μl PBS with one drop Heparin was mixed with two drops of infected blood. The blood-PBS mixture then was injected intravenously into the tail of naïve mice. The development of a patent parasitemia was analyzed by daily examination of Giemsa-stained smears.

### Immunization and parasite challenge experiments

Age-matched female C57BL/6 mice were immunized with 1,000 or 10,000 *Pbsera4(-)*-NK65 sporozoites, injected intravenously. Animals that remained malaria-free after the first immunization were given a second dose. Only animals that remained blood stage parasite-negative after the first immunization and subsequent boost were used for the challenge experiments 2 and 20 months after the last immunization. Mice were challenged by intravenous injection of 10,000 WT sporozoites. At least three age-matched naïve animals were included to verify infectivity of sporozoites during all challenge experiments. Parasitemia was monitored by daily Giemsa-stained thin blood smears, starting from day 3 after immunization or challenge infection until at least day 14.

### Statistical analyses

For assays quantifying parasites, RNA or merosomes, transgenic and wildtype *P*. *berghei* were compared using two-tailed *t*-tests, and details are defined in the figure legends. Assays evaluating time to patency were analyzed using the Mantel-Cox test. All statistical analyses were performed in GraphPad Prism version 7.

## Supporting information

S1 Table Oligonucleotides used in this study. (PDF)

S2 Table Infectivity of *Pbsera4(-)* sporozoites. (PDF)

S3 Table Infectivity of *in vitro*-generated *Pbsera4(-)* merosomes in mice. Huh7 cells in 6-well dishes were infected with *P*. *berghei* sporozoites of the indicated lines, and merosomes and parasite-infected detached cells (collectively referred to as merosomes in table headings) were collected, quantified and the numbers indicated were injected intravenously into naïve NMRI mice.
(PDF)

S4 Table Immunizations of C57BL/6 mice with *Pbsera4(-)*-NK65 sporozoites. Animals were infected intravenously with *Pbsera4(-)*-NK65 sporozoites. Those that remained negative for blood-stage infection were boosted as indicated.
(PDF)

S1 Fig Generation of transgenic *PbSERA4-mCherry* parasites. (A) Control PCRs for detection of *SERA4* and control transcripts. *P*. *berghei* ANKA genomic DNA was used as a template in PCRs run in parallel to those using cDNA as the template shown in Fig 1C. The product sizes are as expected and match those amplified from cDNA. (B) Generation of an endogenously encoded SERA4-mCherry fusion protein using a single cross-over insertion strategy. An integration plasmid containing the region encoding the C-terminus of SERA4 (dark grey box) fused to mCherry (red box) is targeted into the *PbSERA4* genomic locus. The targeting plasmid contains the 3’ UTR of *PbDHFR/TS* (light grey box) and the *Tgdhfr/TS* selectable marker (white box). Integration results in a *PbSERA4* gene that is expressed from its endogenous promoter and tagged with mCherry followed by non-expressed truncated duplication of the 3’ end of the gene. (C) Genotyping of the transgenic *PbSERA4-mCherry* parasite line with the primers indicated in panel B resulted in expected products. (D) Confocal fluorescence microscopy reveals expression of the SERA4-mCherry fusion protein (red) in blood stage parasites, including trophozoites (arrowheads) and gametocytes (asterisk). Scale bars, 8 μm.
(TIF)

S2 Fig Stage-specific *PbSERA*-promoter-driven protein expression. The indicated 5’ regions upstream of the *PbSERA4* or *PbSERA5* coding sequences were cloned in front of GFP into the *P*. *berghei* transfection plasmid pL0031 (right). GFP expression in *P*. *berghei* ANKA parasites transfected with these constructs was monitored across the life cycle stages: midgut-oocysts (oo), salivary gland sporozoites (sp), HepG2 cells infected with transgenic exo-erythrocytic parasites at indicated timepoints (EE) and erythrocytic stage (ES) were stained with Hoechst 33342 and visualized by live microscopy.
(TIF)

S3 Fig Validation of α-*Pb*SERA4M antisera specificity. Recombinant *Pb*SERA proteins (1μg) spanning the central papain-like protease domain (M) were separated on a polyacrylamide gel and stained with Coomassie (top panel). Tags of the MBP-fusion proteins (lane1,2) were cleaved off. The MBP-tag (grey arrow) and resulting proteins (black arrow) are indicated. GST fusion proteins were insoluble and thus were purified by Prep-Cell purification (lane 3, 4) GST-tags could therefore not be cleaved off. Thirty nanograms of these recombinant proteins were run and transferred to a membrane that was subsequently probed with α-*Pb*SERA4M polyclonal antisera. α-*Pb*SERA4M specifically recognized *Pb*SERA4 and not other SERA proteins.
(TIF)

S4 Fig Generation and validation of clonal *Pbsera4(-)* parasites. (A) Experimental strategy for targeted disruption of *PbSERA4*. The wildtype (WT) *PbSERA4* genomic locus is targeted with KpnI/SacII-linearized replacement plasmid containing 5’ and 3’ untranslated regions adjacent to the *SERA4* open reading frames and the *dhfr/ts*-positive selectable marker. Upon a double cross-over homologous recombination, the *PbSERA4* open reading frame is replaced by the selectable marker, resulting in *PbSERA4* knockout parasites termed *Pbsera4(-)*. For genotyping, replacement-specific test and WT test primer combination are indicated by arrows and expected fragments as lines. (B) Genotyping of the *Pbsera4(-)* disruption in *P*. *berghei* ANKA using PCR analysis. The primer combinations that amplify signals in the recombinant locus (test1 and 2) of purified genomic DNA verified the successful replacement event. The absence of a WT specific signal from *Pbsera4(-)* parasites confirms the purity of the clonal populations. (C) *Pb*SERA4 protein is not detected in the *Pbsera4(-)* line. Lysates of mixed-stage infected erythrocytes were analysed by western blot using the anti-*Pb*SERA4M antibody (top). Anti-HSP70 antibodies were used as a loading control (bottom). Full length *Pb*SERA4 and intermediate cleavage products are detected exclusively in the wildtype samples. (D) Genotyping of the *Pbsera4(-)* disruption in *P*. *berghei* NK65 using PCR analysis. The primer combinations that amplify signals in the recombinant locus (test 1 for and test 2 rev) of purified genomic DNA verified the successful replacement event. The absence of a WT specific signal from *Pbsera4(-)-*NK65 parasites confirms the purity of the clonal populations.
(TIF)

S1 Data Spreadsheet containing the numerical data included within Figure panels 3B, 3C, 3E, 3F, 4A and 5B. (XLSX)
