# Supplementary material for: A Plasmodium cysteine protease required for efficient transition from the liver infection stage
Source: PLoS Pathog. 2020 Sep 21;16(9):e1008891. doi: 10.1371/journal.ppat.1008891 (PMC7529260; doi:10.1371/journal.ppat.1008891)
Supplement: S1 Table — (PDF) [file ppat.1008891.s001.pdf]

S1 Table. Oligonucleotides used in this study.

Experiment	Oligonucleotide	Sequence 5' → 3'		
**RT PCR**	*SERA4*_for	CGAAGTAAGAAACAAAGGTTCAG		
	*SERA4*_rev	GGTTGCCAAACAATATCCAGGAGAAGTG		
	*TRAP*_for	CCCGGATCCATGAAGCTCTTAGGAAATAG		
	*TRAP*_rev	GTGTGGATCCTTCCTGACAAACTTTAGAAAG		
	*GAPDH*_for	ATGGCAATAACAAAAGTCGGAATTAATGG		
	*GAPDH*_rev	TGTGGATAGCCAAATCTAAAAGACGG		
	*MSP1*_for	CTGGTTTGGTAGGAGAAGGCGAATC		
	*MSP1*_rev	AGCTACAGAATACACCATCATAAT		
**qPCR**	*Pb18S_for*	AAGCATTAAATAAAGCGAATACATCCTTAC		
	Pb18S_rev	GGAGATTGGTTTTGACGTTTATGTG		
	mHPRT for	TGCTCGAGATGTGATGAAGG		
	mHPRT rev	TCCCCTGTTGACTGGTCATT		
	SERA3_for	GTTGATGTTTTAGGTCCAGATAATTGTG		
	SERA3_rev	GTGGTAAAAATTTGAACTGAGTTGTGG		
***SERA4* targeting vectors**	mCh-SERA4for	ATAAGAATGCGGCCGCACGAGATTGACAATGAAAATGAAATAATGG		
	mCh-SERA4rev	TGCTCTAGATATGCTGCAAAAGAAACAATCTTCACC		
	SERA4rep1for	GGGGTACCCACAATTGAAGAGGAATGACGAAAAAGGA		
	SERA4rep2rev	GCCCAAGCTTCATTGGGTGGGTTTGAGGTTTGCGT		
	SERA4rep3for	CGCGGATCCGTACGTGGATAGGAAACTTTTAAAGGTA		
	SERA4rep4rev	TCCCCGCGGCCCAACCCAATGATAAGTACAGTCATGCA		
**Tests for selected parasites**	Sera4tag_for	ATATGAAAAGCTTAGAAACCCAGC		
	Sera4tag_rev	TACCTTTAAAAGTTTCCTATCCACG		
	Mcherry_rev	GATCCTTACTTGTACAGC		
	T7	GTAATACGACTCACTATAGGGC		
	Sera4test_for	ACATTTAGCGCATGTTGAATGCTTATACAGC		
	DHFR_rev	CGCATTATATGAGTTCATTTTACACAATCC		
	Sera4test_rev	GGATCCATACCTTGATTTATCAGATTGAT		
	DHFR_for	CCCGCACGGACGAATCCAGATGG		
***PbSERA*-promoter-driven GFP expression**	SERA4-5´for	TCCCCGCGGTGGGAACGCTGTAAAAATCA		
	SERA4-5´rev	CATGCCATGGTTTGTTACAATAATTTTAATCTTGAAACATTTTACTTCA		
	SERA5-5´for	CGTCTAGACGGCTTGTTTAGTAATTTATAACT		
	SERA5-5´rev	TATCCATGGCTTCCGTTATTTTTAAAAGTTTGTC		
			protein	tag
**Recombi-nant protein expression**	SERA4-M-for	TGTGGATCCTTCACAGCAAATGCAAAAGAA	Phe$_{216}$-His$_{365}$	GST
	SERA4-M-rev	TGTCTCGAGCATGAGCATCTAATGTGCCACT		
	SERA4-N-for	TTGAATTCCACGAAATTAATACGCAAACCT	His$_{20}$-Asn$_{188}$	GST
	SERA4-N-rev	GAATGCGGCCGCTCATTAGTGTGTGTTTCCCATT		
	SERA1-M-for	TTCGGATCCTATTGCAATGATGAATATTGTGACAG	Tyr$_{586}$-Tyr$_{869}$	MBP
	SERA1-M-rev	AACTGCAGTCAGTAAAAATCAGGAGATATTTTTGAA		
	SERA2-M-for	TGAATTCAAACATGTAGAACAATGGGTTGC	Lys$_{546}$-Pro$_{714}$	GST
	SERA2-M-rev	TGTGGCGGCCGCTATGGATAATTGGATTCAGTTGG		
	SERA3-M-for	CGGGATCCAAAGTAACTGCTCAAAGTGATGAAGA	Trp$_{620}$-Ser$_{875}$	MBP
	SERA3-M-rev	AACTGCAGAATTGAATCACTATCTGGCTCCAT		

Restriction sites are underlined. For recombinant proteins production, the amino acid position of the respective *Pb*SERA proteins and the fusion tag for subsequent purification (MBP or GST) are indicated.
