# Supplementary material for: A Plasmodium cysteine protease required for efficient transition from the liver infection stage
Source: PLoS Pathog. 2020 Sep 21;16(9):e1008891. doi: 10.1371/journal.ppat.1008891 (PMC7529260; doi:10.1371/journal.ppat.1008891)
Supplement: S2 Table — (PDF) [file ppat.1008891.s002.pdf]

S2 Table. Infectivity of *Pbsera4(-)* sporozoites.

Sporozoite dose	Parasite line	No. infected animals/ No. injected animals		Time to patency (days)	
		SD Rat	C57BL/6	SD Rat	C57BL/6
1,000	wildtype-NK65	2/2	4/4	3.0	3.5
	Pbsera4(-)-NK65	2/2	3/13	7.0	(8.3)
10,000	wildtype-ANKA	9/9	10/10[a]	3.0	3.5[a]
	Pbsera4(-)-ANKA	11/13	10/10[a]	(5.1)	5.0[a]
	wildtype-NK65	5/5	8/8[b]	3.3	3.5[b]
	Pbsera4(-)-NK65	3/4	15/26[b]	(7.0)	(7.6)[b]
100,000	wildtype-ANKA	1/1	3/3	3.0	3.0
	Pbsera4(-)-ANKA	1/1	3/3	5.0	4.6
mosquito bite	wildtype-NK65	2/2	2/2	3.0	3.0
	Pbsera4(-)-NK65	2/2	2/2	7.0	7.0

No. infected animals/No. injected animals indicates the number of animals in which blood-stage infection was detected relative to the number that were injected with the indicated numbers of sporozoites.

Time to patency is the mean number of days between infection and detection of blood-stage *P. berghei.* Parentheses indicate that not all animals became blood smear-positive.

[a] The data are displayed in Figure 3D.

[b] The data are displayed in Figure 5A.
