# Supplementary material for: A Plasmodium cysteine protease required for efficient transition from the liver infection stage
Source: PLoS Pathog. 2020 Sep 21;16(9):e1008891. doi: 10.1371/journal.ppat.1008891 (PMC7529260; doi:10.1371/journal.ppat.1008891)
Supplement: S3 Table — Huh7 cells in 6-well dishes were infected with P. berghei sporozoites of the indicated lines, and merosomes and parasite-infected detached cells (collectively referred to as merosomes in table headings) were collected, quantified and the numbers indicated were injected intravenously into naïve NMRI mice. (PDF) [file ppat.1008891.s003.pdf]

S3 Table. Infectivity of *in vitro*-generated *Pbsera4(-)* merosomes in mice

Exp.	Parasite line	Average no. merosomes released per well	No. merosomes injected per animal	No. infected animals/no. injected animals	Time to patency (days)
I	wildtype-ANKA	5,360	500	3/3	4.0
	Pbsera4(-)-ANKA	2,350	500	3/3	4.6
II	wildtype-ANKA	3,100	500	3/3	4.3
	Pbsera4(-)-ANKA	430	500	3/3	5.0
III	wildtype-ANKA	2,700	2,700	1/1	3.0
	Pbsera4(-)-ANKA	1,930	1930	1/1	3.0
IV	wildtype-ANKA	650	650	2/2	6.0
	Pbsera4(-)-ANKA	550	550	2/2	6.0
V	wildtype-ANKA	200	100	2/2	5.0
	Pbsera4(-)-ANKA	46	92	2/2	5.0

Exp. indicates separate experiments.

The data in column 3 (Average no. merosomes released per well) are displayed graphically in Figure 4C where the values are normalized to wildtype-ANKA.

No. infected animals/No. injected animals indicates the number of animals in which blood-stage infection was detected relative to the number that were injected with merosomes.

Time to patency is the mean number of days between infection and detection of blood-stage *P. berghei.*
