# Supplementary material for: A Plasmodium cysteine protease required for efficient transition from the liver infection stage
Source: PLoS Pathog. 2020 Sep 21;16(9):e1008891. doi: 10.1371/journal.ppat.1008891 (PMC7529260; doi:10.1371/journal.ppat.1008891)
Supplement: S4 Table — Animals were infected intravenously with Pbsera4(-)-NK65 sporozoites. Those that remained negative for blood-stage infection were boosted as indicated. (PDF) [file ppat.1008891.s004.pdf]

S4 Table. Immunizations of C57BL/6 mice with *Pbsera4(-)*-NK65 sporozoites

No. of *Pbsera4(-)*-NK65 sporozoites injected for immunizations	Boost (day after initial immunization / no. of sporozoites	No. protected / No. challenged		Protection (%) 2 months / 20months
		2 months since last immunization	20 months since last immunization	
**10,000**	1st(14/10,000), 2nd(28/10,000)	5/5	0/5	**100/0**
	1st(14/10,000), 2nd(28/10,000), WT challenge (89/10,000)	n.a.	5/5	n.a./**100**
**naive**	--	0/3	0/3	**0/0**
**1,000**	1st(14/1,000), 2nd(28/1,000)	1/4	0/6	**25/0**
	1st(14/1,000), 2nd(28/1,000), WT challenge (89/10,000)	n.a.	0/1	n.a./**0**
**naive**	--	0/2	0/3	**0/0**

"WT challenge" indicates that animals received a dose of 10,000 wildtype sporozoites at the indicated time-point and remained blood-stage negative (these are the protected animals in the preceding row). No. protected / No. challenged indicates the number of animals that did not develop a blood-stage infection of those that received the challenge dose of 10,000 wildtype sporozoites.
