# Supplementary figures and images for: A Plasmodium cysteine protease required for efficient transition from the liver infection stage
Source: PLoS Pathog. 2020 Sep 21;16(9):e1008891. doi: 10.1371/journal.ppat.1008891 (PMC7529260; doi:10.1371/journal.ppat.1008891)

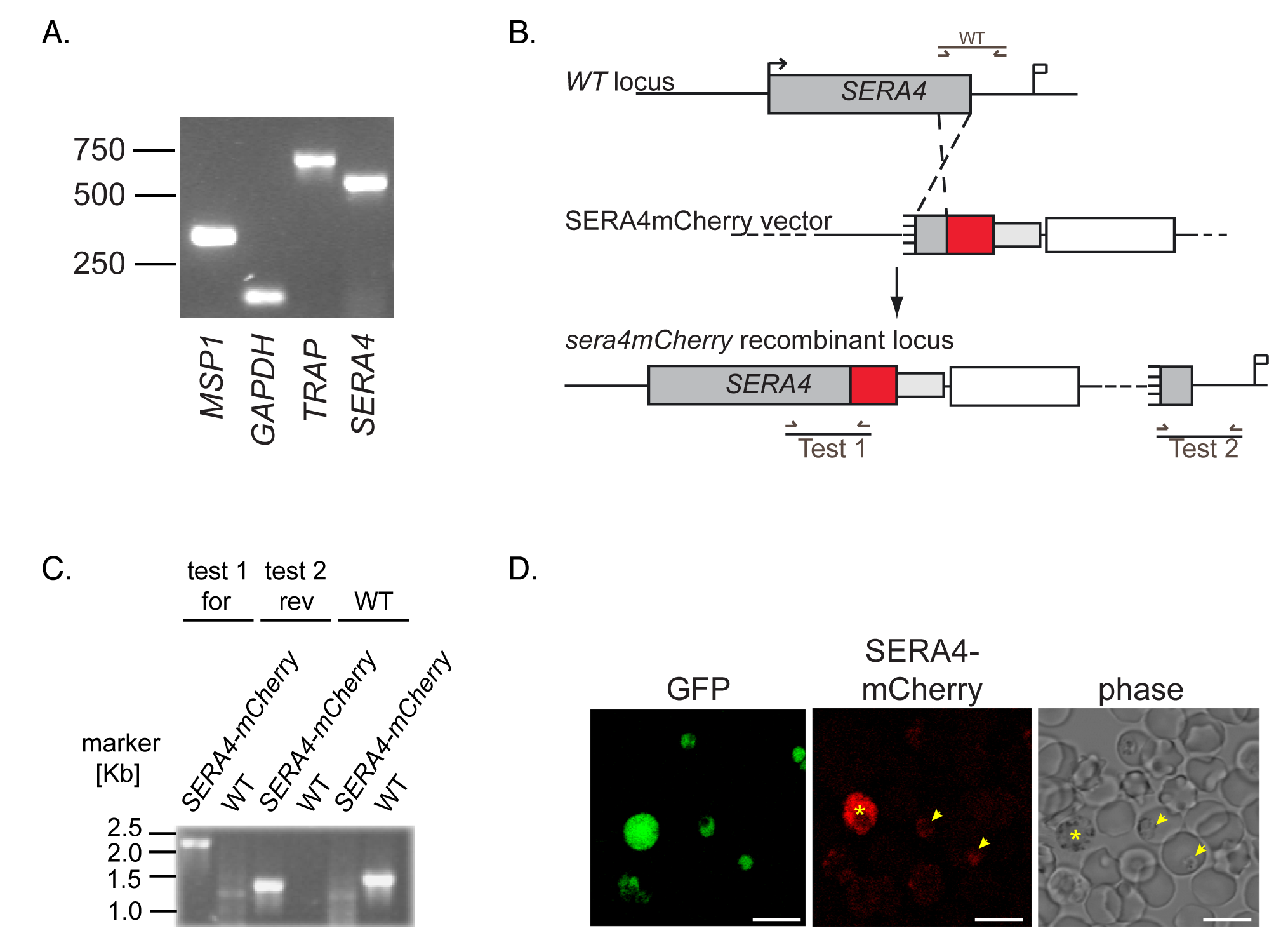

Supplement: S1 Fig — (A) Control PCRs for detection of SERA4 and control transcripts. P. berghei ANKA genomic DNA was used as a template in PCRs run in parallel to those using cDNA as the template shown in Fig 1C. The product sizes are as expected and match those amplified from cDNA. (B) Generation of an endogenously encoded SERA4-mCherry fusion protein using a single cross-over insertion strategy. An integration plasmid containing the region encoding the C-terminus of SERA4 (dark grey box) fused to mCherry (red box) is targeted into the PbSERA4 genomic locus. The targeting plasmid contains the 3’ UTR of PbDHFR/TS (light grey box) and the Tgdhfr/TS selectable marker (white box). Integration results in a PbSERA4 gene that is expressed from its endogenous promoter and tagged with mCherry followed by non-expressed truncated duplication of the 3’ end of the gene. (C) Genotyping of the transgenic PbSERA4-mCherry parasite line with the primers indicated in panel B resulted in expected products. (D) Confocal fluorescence microscopy reveals expression of the SERA4-mCherry fusion protein (red) in blood stage parasites, including trophozoites (arrowheads) and gametocytes (asterisk). Scale bars, 8 μm. (TIF) [file ppat.1008891.s005.tif]

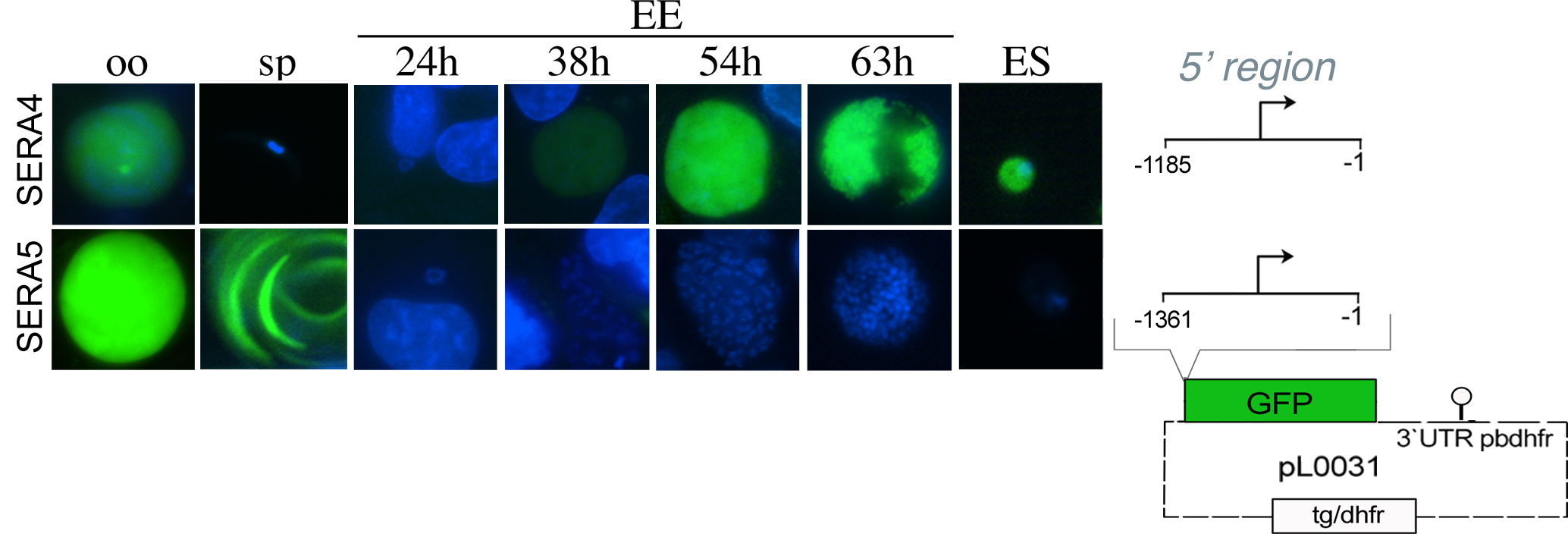

Supplement: S2 Fig — The indicated 5’ regions upstream of the PbSERA4 or PbSERA5 coding sequences were cloned in front of GFP into the P. berghei transfection plasmid pL0031 (right). GFP expression in P. berghei ANKA parasites transfected with these constructs was monitored across the life cycle stages: midgut-oocysts (oo), salivary gland sporozoites (sp), HepG2 cells infected with transgenic exo-erythrocytic parasites at indicated timepoints (EE) and erythrocytic stage (ES) were stained with Hoechst 33342 and visualized by live microscopy. (TIF) [file ppat.1008891.s006.tif]

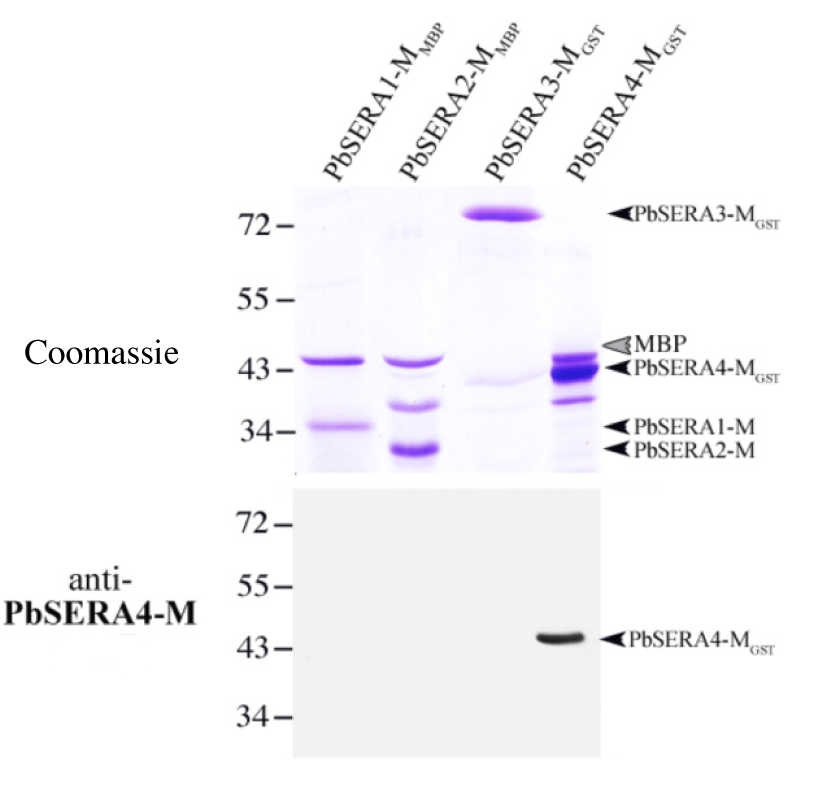

Supplement: S3 Fig — Recombinant PbSERA proteins (1μg) spanning the central papain-like protease domain (M) were separated on a polyacrylamide gel and stained with Coomassie (top panel). Tags of the MBP-fusion proteins (lane1,2) were cleaved off. The MBP-tag (grey arrow) and resulting proteins (black arrow) are indicated. GST fusion proteins were insoluble and thus were purified by Prep-Cell purification (lane 3, 4) GST-tags could therefore not be cleaved off. Thirty nanograms of these recombinant proteins were run and transferred to a membrane that was subsequently probed with α-PbSERA4M polyclonal antisera. α-PbSERA4M specifically recognized PbSERA4 and not other SERA proteins. (TIF) [file ppat.1008891.s007.tif]

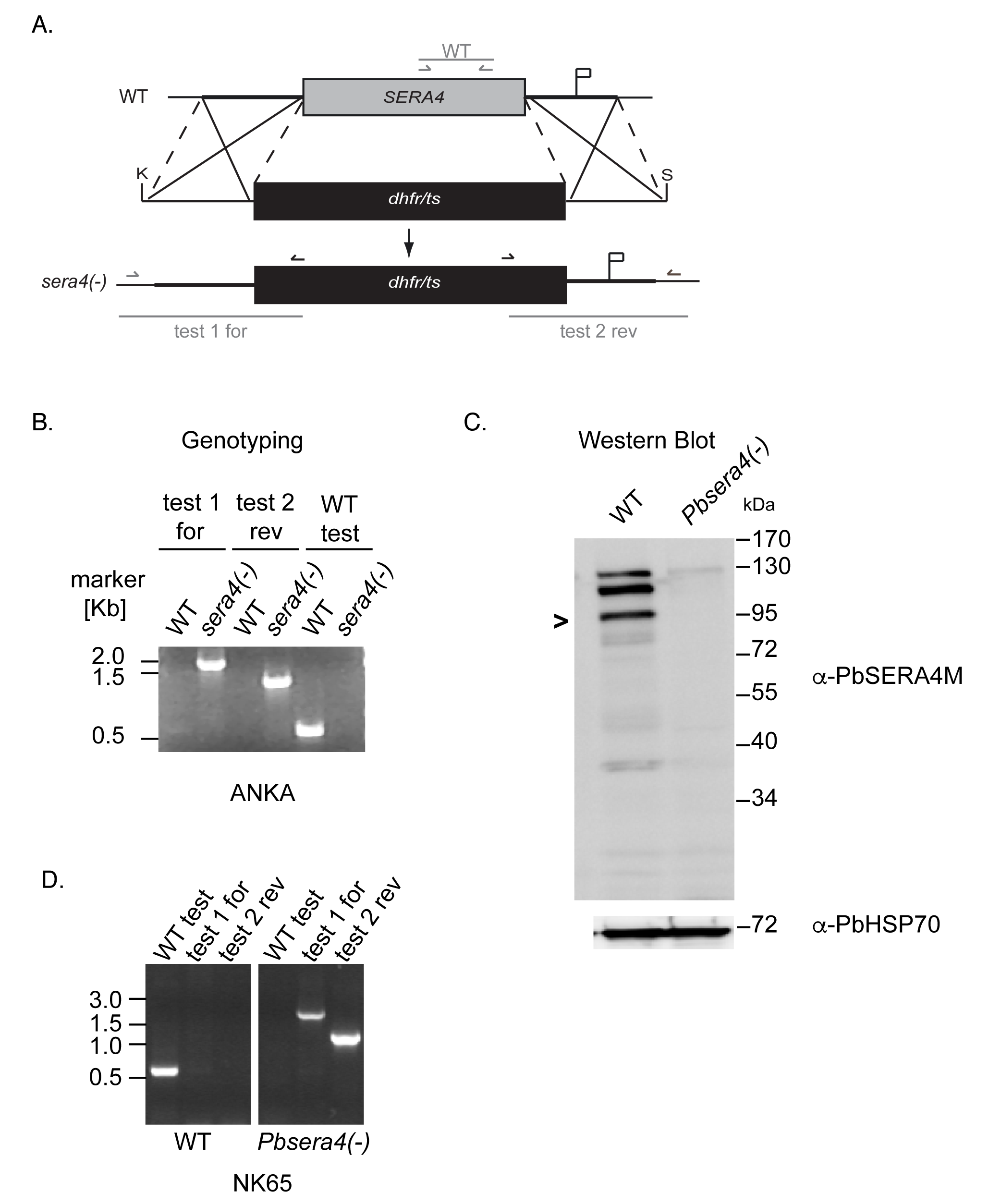

Supplement: S4 Fig — (A) Experimental strategy for targeted disruption of PbSERA4. The wildtype (WT) PbSERA4 genomic locus is targeted with KpnI/SacII-linearized replacement plasmid containing 5’ and 3’ untranslated regions adjacent to the SERA4 open reading frames and the dhfr/ts-positive selectable marker. Upon a double cross-over homologous recombination, the PbSERA4 open reading frame is replaced by the selectable marker, resulting in PbSERA4 knockout parasites termed Pbsera4(-). For genotyping, replacement-specific test and WT test primer combination are indicated by arrows and expected fragments as lines. (B) Genotyping of the Pbsera4(-) disruption in P. berghei ANKA using PCR analysis. The primer combinations that amplify signals in the recombinant locus (test1 and 2) of purified genomic DNA verified the successful replacement event. The absence of a WT specific signal from Pbsera4(-) parasites confirms the purity of the clonal populations. (C) PbSERA4 protein is not detected in the Pbsera4(-) line. Lysates of mixed-stage infected erythrocytes were analysed by western blot using the anti-PbSERA4M antibody (top). Anti-HSP70 antibodies were used as a loading control (bottom). Full length PbSERA4 and intermediate cleavage products are detected exclusively in the wildtype samples. (D) Genotyping of the Pbsera4(-) disruption in P. berghei NK65 using PCR analysis. The primer combinations that amplify signals in the recombinant locus (test 1 for and test 2 rev) of purified genomic DNA verified the successful replacement event. The absence of a WT specific signal from Pbsera4(-)-NK65 parasites confirms the purity of the clonal populations. (TIF) [file ppat.1008891.s008.tif]
